# Improvements in life expectancy among Australians due to reductions in smoking: Results from a risk percentiles approach

**DOI:** 10.1186/s12889-016-2750-5

**Published:** 2016-01-26

**Authors:** Haider Mannan, Andrea J Curtis, Andrew Forbes, Dianna J Maglianno, Judy A Lowthian, Manoj Gambhir, John J McNeil

**Affiliations:** 1 Centre for Health Research, School of Medicine, Western Sydney University, Building 3, level G, Campbelltown campus, NSW 2560 Australia; 2 Department of Epidemiology & Preventive Medicine, Monash University, level 3, Alfred Centre, 99 Commercial Road, Melbourne, Victoria 3004 Australia; 3 Baker International Diabetes Institute, Melbourne, Victoria 3805 Australia

**Keywords:** Smoking, Reduction, Life expectancy, Australia, Risk percentiles

## Abstract

**Background:**

Tobacco smoking is a major burden on the Australian population in terms of health, social and economic costs. Because of this, in 2008, all Australian Governments agreed to set targets to reduce prevalence of smoking to 10 % by 2018 and subsequently introduced several very strong anti-smoking measures. On this backdrop, we estimated in 2012-13 the impact of several scenarios related to reduction of smoking prevalence to 10 % across the entire Australian population and for below specific ages, on improving life expectancy.

**Methods:**

Using the risk percentiles method the Australian Diabetes, Obesity and Lifestyle (AUSDIAB) baseline survey and the Australian Bureau of Statistics (ABS) age-sex specific death counts were analyzed.

**Results:**

Amongst men the gains in life expectancy associated with 10 % smoking prevalence are generally greater than those of women with average life expectancy for men increasing by 0.11 to 0.41 years, and for women by 0.12 to 0.29 years. These are at best 54 % and 49 % for men and women of the gains achieved by complete smoking cessation. The gains plateau for interventions targeting those <70 and <80 years. Amongst smokers the potential gains are much greater, with an increase in average life expectancy amongst men smokers of 0.43 to 2.08 years, and 0.73 to 2.05 years amongst women smokers. These are at best 46 % and 38 % for men and women smokers of the gains achieved by complete smoking cessation.

**Conclusion:**

The estimated optimum gain in life expectancy is consistent with potentially moderate gains which occur when both men and women below 60 years are targeted to reduce smoking prevalence to 10 %.

**Electronic supplementary material:**

The online version of this article (doi:10.1186/s12889-016-2750-5) contains supplementary material, which is available to authorized users.

## What this paper adds?

This is the first study in Australia evaluating the impact of the smoking reduction target set by various Australian governments by 2018, on improving life expectancy. It achieves this objective by estimating the magnitude of gain in life expectancy if the smoking reduction target is met and by how much this accounts for the maximum gain in life expectancy if complete smoking cessation is achieved. This study provides direction on how to better target the Australian adult population by their basic demographics such as age and gender rather than by only targeting the entire adult population ignoring their age and gender. In view of its importance to smoking control and its likely impact on health in general, the paper is important for assessing the role of tobacco control policies on improvements in general health and wellbeing. It discusses whether the set target is achievable by 2018 and points out to further research in terms of the impact on survival with further reduction in smoking than has been targeted.

## What is already known?

It is already known that the smoking prevalence has been targeted to reduce to 10 percent by 2018 in Australia. It is also known that this will improve average life expectancy of the Australians.

### Highlights

● Amongst men the potential gains in life expectancy associated with 10 % smoking prevalence are greater than those of women, likely due to the higher baseline prevalence in men.

● The maximal gain in life expectancy for reducing smoking prevalence to 10 % at the population level occurred when men and women below 60 years were targeted.

● These are at best 54 % and 49 % for men and women respectively of the life expectancy gains achieved by complete smoking cessation in the population

● These are at best 46 % and 38 % for men and women smokers.

## Background

Tobacco smoking is a major burden on the Australian community in terms of health, social and economic costs. Smoking contributes to the development of all major chronic diseases, including heart disease and stroke, cancer, and respiratory disease. It is the largest cause of premature death in Australia, contributing to an estimated 15,000 deaths each year [1, 2]. In addition to the direct costs associated with provision of care for smoking related illnesses, additional costs to the community include loss of productivity due to absenteeism or reduction in the workforce resulting from premature death. Collins and Lapsley [1] estimated the total cost of smoking to the Australian community to be $31.5 billion in 2004-5. This represented an increase of 23.5 % over five years, in the total costs estimated for 1998-99.

In 2008, through the Council of Australian Governments (COAG) National Healthcare Agreement and the National Partnership Agreement on Preventive Health [3], all Australian Governments agreed to set targets to reduce the prevalence of adult daily smoking to 10 % by 2018, and to halve the rate of smoking amongst Torres Strait Islander and Aboriginal people. To achieve this target, the Australian government has introduced, among others, plain packaging, graphic health warnings on cigarette packages, 25 % tobacco excise increase in April 2010 and record investment in anti-smoking social marketing campaigns – one of the strongest measures taken anywhere else in the developed world to curb cigarette smoking. The 2009 National Preventive Health Strategy [4] set out the actions recommended by the National Preventive Taskforce to achieve these goals. The National Tobacco Strategy 2012-2018 [5] takes account of these key strategies and sets out a policy framework. The health impact of achieving these Government targets for smoking prevalence is unknown.

Smoking prevalence in Australia varies across age groups. In general, smoking prevalence decreases with increasing age. In 2011-2012, Australians in the 25-34 year age group exhibited the highest rates for current smoking with 23.9 % being current smokers. This was followed by the age group 35-44 with 20.4 % being current smokers. During the same period, 8.9 % of those aged 65-74 were current smokers compared with 21.7 % in the 45-54 year age group and 15.1 % in the 55-64 year age group. Amongst younger Australians, smoking prevalence was 6.6 % for those aged 15-17 years and 19.5 % for those aged 18-24 years [6].

In the context of the National Tobacco Strategy target to reduce smoking prevalence in Australia to 10 % by 2018, this study aimed to model the impact of smoking reduction on improvements in average life expectancies in the Australian population. Although the prevalence of smoking declines with age, the impact of targeting specific age groups for reduction of smoking prevalence in terms of maximal impact on life expectancy is unknown. In view of this we modelled scenarios in which smoking prevalence were reduced to 10 % across the entire population and below certain ages and estimated the impact on life expectancy. All analyses were performed separately for men and women. This paper has important health implications for Australia and because of the novelty of setting national targets on smoking reduction, has implications globally particularly for other developed countries where such national targets are yet to be set.

## Methods

We briefly describe the risk percentiles methodology which is adapted from an earlier coronary heart disease (CHD) prevention model [7]. Based on the information from the baseline survey of the AUSDIAB study (described below) which we used in this study, an all-cause mortality risk equation, the European Cardiovascular SCORE or EURO SCORE [8] was applied to each individual and these were then ordered to generate percentiles of mortality risk. The EURO SCORE risk equation considered a binary categorization for smoking status, namely, ‘current smoker’ and ‘non-smoker’ and hence did not distinguish between ex-smoker and never smoker.

The overall population was evenly spread between the percentiles of mortality risk; however the absolute risk was higher in higher percentiles and vice versa. The observed counts of deaths based on the national death numbers were then allocated to each percentile of mortality risk in proportion to the modelled level of absolute risk in each percentile, enabling mortality rates to be calculated for each percentile of risk. Using these mortality rates life tables were then constructed for people at different percentiles or levels of risk, producing percentile-risk-specific survival curves describing the survival experience (life expectancy) associated with each risk level. Averaging these life expectancies over the population results in the population average life expectancy (ALE). We term this the “Base Scenario”, meaning the ALE in the population with its existing risk profile. For any hypothetical intervention to reduce smoking prevalence there will be a resulting change in the overall population risk factor profile due to a certain proportion of smokers becoming non-smokers which was obtained through Monte Carlo simulation. Such individuals would then have reduced risk, and their life expectancy altered according to this reduced risk. Averaging over the population produces the average life expectancy associated with the particular smoking intervention. The average life expectancy for each intervention was then compared with that of the “Base Scenario” to determine the impact of the intervention at a population level. Further details of this method and the calculations of ALE are provided in Additional file 1. Briefly, the steps for risk percentiles method are:

- Estimate risk scores for mortality for every individual in our study cohort using the EURO SCORE.
- Divide the Australian population into mortality risk percentiles using these risk scores.
- Use the ratios of the risk scores to allocate deaths in the Australian population to risk percentiles.
- Divide these death counts by the age group-sex specific Australian population to give us the mortality rate for each risk percentile group within each sex and age group.
- Use these mortality rates to construct sex-specific life tables for each risk percentile within each sex group.
- Calculate a baseline average life expectancy (ALE) per person for each sex.
- Use bootstrapping to construct confidence intervals for ALE
- Change a certain percentage of current smokers to non-smokers.
- Re-allocate participants to risk percentiles.
- Calculate the scenario ALE.
- Derive the effect of reductions in smoking prevalence on mortality by calculating the gain in life expectancy.
- Use bootstrapping to construct confidence intervals of scenario ALE.

The EURO SCORE calibrates well to the Australian population based on the baseline survey of the AUSDIAB study [9]. Although this risk equation was developed for those aged ≥40 years, we applied this to a sample aged ≥25 years, which is valid under the assumption that the ordering of risk in the age group 25-39 follows that of the ordering of risk based on EURO SCORE.

The principal scenario modelled the impact on ALE of achieving the National targets for smoking prevalence, that is, a reduction from existing levels to 10 % by 2018. A range of hypothetical intervention scenarios were chosen to demonstrate the impact of targeting reductions in smoking prevalence in specific age groups. Once the gains in life expectancy for the interventions were estimated at the population level through the risk percentiles approach, they were approximated for the smokers (the method is described in Additional file 1). Table 1 summarises the smoking intervention scenarios investigated using this model.

**Table 1:** Smoking Prevalence Scenarios Modelled With Mortality Projections (study period 2012-13)

Scenario	Targeted Population	Change in smoking behaviour
Base scenario	Whole population	No change
0 % prevalence <30 years	25-29 year age groups	All smokers in targeted age group quit
10 % prevalence <30 years	25-29 year age groups	Prevalence reduced to 10 % for both men and women aged <30 years
0 % prevalence <40 years	25-39 year age groups	All smokers in targeted age group quit
10 % prevalence <40 years	25-39 year age groups	Prevalence reduced to 10 % for both men and women aged <40 years
0 % prevalence <50 years	25-49 year age groups	All smokers in targeted age group quit
10 % prevalence <50 years	25-49 year age groups	Prevalence reduced to 10 % for both men and women aged <50 years
0 % prevalence <60 years	25-59 year age groups	All smokers in targeted age group quit
10 % prevalence <60 years	25-59 year age groups	Prevalence reduced to 10 % for both men and women aged <60 years
0 % prevalence <70 years	25-69 year age groups	All smokers in targeted age group quit
10 % prevalence <70 years	25-69 year age groups	Prevalence reduced to 10 % for both men and women aged <70 years
0 % prevalence <80 years	25-79 year age groups	All smokers in targeted age group quit
10 % prevalence <80 years	25-79 year age groups	Prevalence reduced to 10 % for both men and women aged <80 years
10 % prevalence in entire population	Whole population	Prevalence randomly reduced to 10 % for both men and women
0 % prevalence in entire population	Whole population	All smokers quit

The baseline survey of the AUSDIAB study was a cross-sectional, national, population-based survey conducted in 1999–2000 [10]. In this survey, 11,190 participants (5,483 men and 5,707 women) aged > = 25 years had complete data for a number of cardiovascular risk factors including systolic and diastolic blood pressure, self-reported cigarette smoking status, and serum total cholesterol.

We obtained national population and death counts classified by age and sex for the years 2001 to 2006 from the Australian Institute of Health and Welfare (AIHW). The population counts were derived from the Australian Bureau of Statistics (ABS) mid-year population estimates [11]. Death data was derived from the AIHW National Mortality Database comprising all deaths registered in Australia.

## Results

In AUSDIAB the overall prevalence of current smoking was 18.73 % for men and 13.16 % for women. Table 2 shows estimates of the national prevalence of current smoking and number of (current) smokers by age and sex obtained from the AUSDIAB baseline examination [12].

**Table 2:** National Prevalence of Smoking in Australia as Estimated From the AUSDIAB Baseline Examination (study period 2012-13)

Age group	Prevalence	Smokers	Prevalence	Smokers
	(Men)	(Men)	(Women)	(Women)
25-29	25.60	175987	16.47	93153
30-34	22.30	166508	19.74	170786
35-39	26.07	172920	19.21	117507
40-44	21.24	165277	14.58	122826
45-49	19.58	116580	14.33	90297
50-54	19.21	123686	13.59	79154
55-59	15.97	72234	9.25	40025
60-64	11.06	39408	9.85	35656
65-69	12.02	44849	6.09	25581
70-74	5.77	17481	5.89	23537
75-79	4.70	8737	3.03	8472
80-84	5.07	5310	3.68	5172
85+	1.19	374	0.14	55
Total	18.73	1109351	13.16	812221

Table 3 shows the number of people who have to quit smoking under each intervention scenario based on the AUSDIAB baseline examination [12]. The number of quitters increases disproportionately with age and slows down after <60 years for men and after <50 years for women.

**Table 3:** Estimated number of quitters to achieve each target scenario (study period 2012-13)

Scenario	Men	Women
Base scenario	___	___
0 % prevalence <30 years	175987	93153
10 % prevalence <30 years	107242	36594
0 % prevalence <40 years	515415	381446
10 % prevalence <40 years	305690	177212
0 % prevalence <50 years	797272	594569
10 % prevalence <50 years	450180	243064
0 % prevalence <60 years	993192	713748
10 % prevalence <60 years	536484	260724
0 % prevalence <70 years	1077448	774985
10 % prevalence <70 years	547770	243777
0 % prevalence <80 years	1103666	806994
10 % prevalence <80 years	525108	207900
10 % prevalence in entire population	517065	195032
0 % prevalence in entire population	1109351	812221

Table 4 shows the estimated mortality risks for the smokers and nonsmokers (separately for men and women) for all interventions if the smoking prevalence and other risk factors during the baseline examination remained unchanged. It shows that the mortality risks for smokers are higher than nonsmokers in all interventions. Men have higher mortality risks than women.

**Table 4:** Mortality risks per 100,000 people by smoking status and sex for the interventions before any smoking reduction is achieved using the AUSDIAB Baseline Examination (study period 2012-13)

Age group	Current Smokers	Non-smokers	Current Smokers	Non-smokers
	(Men)	(Men)	(Women)	(Women)
<30	85	40	4	2
<40	414	211	37	21
<50	1288	754	181	116
<60	2748	1749	608	419
<70	4520	3298	1200	1126
<80	5619	5417	2445	1981
All ages	6348	6009	3227	2242

Tables 5 and 6 present the ALE and the gain in ALE relative to the Base Scenario for each of the modelled scenarios for women and men respectively. In comparison to the Base Scenario there is a gain in ALE regardless of the age at which people stop smoking.

**Table 5:** Gain in Average Life Expectancy (ALE) in the Whole Population Under Various Smoking Reduction Scenarios, for Women (study period 2012-13)

Scenarios	ALE (years)		Gain in ALE relative to Base scenario (years)	
	Women	95 % CI	Women	95 % CI
Base scenario	33.16	32.82,33.50	-	-
0 % prevalence <30 years	33.31	32.95, 33.69	0.15	0.13,0.17
10 % prevalence <30 years*	33.28	32.92, 33.65	0.12	0.10,0.15
0 % prevalence <40 years	33.50	33.12, 33.85	0.34	0.30,0.36
10 % prevalence <40 years	33.38	33.00, 33.71	0.21	0.18,0.22
0 % prevalence <50 years	33.65	33.23,34.28	0.48	0.45,0.5
10 % prevalence <50 years	33.45	33.07, 33.78	0.26	0.24,0.28
0 % prevalence <60 years	33.75	33.34,34.18	0.59	0.51,0.68
10 % prevalence <60 years	33.45	33.09, 33.81	0.29	0.27,0.31
0 % prevalence <70 years	33.83	33.44,34.22	0.66	0.62,0.72
10 % prevalence <70 years	33.45	33.09, 33.81	0.29	0.27,0.31
0 % prevalence <80 years	33.88	33.54,34.22	0.71	0.7,0.72
10 % prevalence <80 years	33.44	33.07, 33.78	0.27	0.25,0.29
10 % prevalence in entire population	33.43	33.41, 33.47	0.27	−0.03 ,0.59
0 % prevalence in entire population	33.90	33.54,34.24	0.73	0.72,0.74

**Table 6:** Gain in Average Life Expectancy (ALE) in the Whole Population Under Various Smoking Reduction Scenarios, for Men (study period 2012-13)

Scenarios	ALE (years)		Gain in ALE relative to Base scenario (years)	
	Men	95 % CI	Men	95 % CI
Base scenario	29.08	28.77,29.39	-	-
0 % prevalence <30 years	29.23	28.86, 29.53	0.16	-.17,.44
10 % prevalence <30 years*	29.19	28.89, 29.50	0.11	0.10,0.12
0 % prevalence <40 years	29.48	29.15,29.79	0.40	0.36,0.43
10 % prevalence <40 years	29.34	29.02, 29.65	0.26	0.25,0.27
0 % prevalence <50 years	29.67	29.34,30.01	0.6	0.57,0.62
10 % prevalence <50 years	29.43	29.10, 29.75	0.358	0.330.36
0 % prevalence <60 years	29.82	29.18,30.46	0.74	0.62,0.82
10 % prevalence <60 years	29.48	29.16, 29.81	0.4	0.39,0.42
0 % prevalence <70 years	29.90	29.55,30.25	0.82	0.78,0.86
10 % prevalence <70 years	29.49	29.11, 29.87	0.41	0.1,0.82
0 % prevalence <80 years	29.92	29.47,30.18	0.85	0.8 ,0.89
10 % prevalence <80 years	29.47	29.09, 29.85	0.39	0.12,0.66
10 % prevalence in entire population	29.46	29.14, 29.81	0.39	0.36,0.43
0 % prevalence in entire population	29.94	29.62,30.39	0.86	0.72,1.0

The maximum gain in ALE occurs when everyone in the population ceases smoking (0 % prevalence). The magnitude of this gain is 0.86 and 0.73 years for men and women, respectively. The gain in ALE increases as more of the older population is targeted, for both a target prevalence of 0 % and 10 %, respectively. However, the additional gain in ALE for an intervention to achieve a smoking prevalence target of 10 % is minimal when persons at least age 60 are included in the intervention. The maximal gain in ALE for men when prevalence in each age intervention group is reduced to 10 % is approximately 54 % of the corresponding gain when smoking is eliminated from the entire population, and for women it is approximately 49 %.

**Fig. 1:**
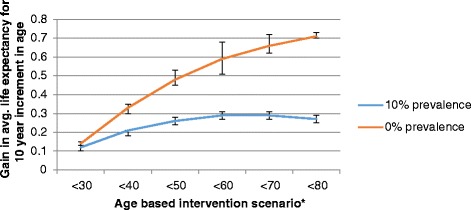
Overall gain in average life expectancy (ALE) for all women by intervention scenario. *For example, <40 indicates intervention to reduced smoking for all individuals aged <40 years

Figure 1 depicts the gain in ALE results for women summarised in Table 5. It presents the overall gain in ALE by intervention scenario. For scenarios in which smoking prevalence is reduced to 0 %, the smallest gain in ALE occurs when only women less than 30 years are targeted. The gain in ALE initially increases as older age groups are included in the interventions; however the effect flattens when all individuals aged <70 years are included. In comparison, for scenarios in which smoking prevalence is reduced to 10 %, the gain in ALE essentially plateaus for interventions targeting additional individuals aged at least 60 years. Specifically, when smoking prevalence is reduced to 10 % amongst women aged <60 years, the gain in ALE is 0.29 years which is about half of the maximum gain achievable through complete cessation for that specific intervention. If women between 60 and 80 years are included in the targeted population, there are no further gains in ALE. If smoking is eliminated amongst all women the gain in ALE is 0.73 years. In comparison, a reduction in smoking prevalence to 10 % amongst all women realizes an ALE gain of 0.27 years, i.e., reducing smoking prevalence to 10 % achieves only 37 % of the maximum possible gain in ALE.

**Fig. 2:**
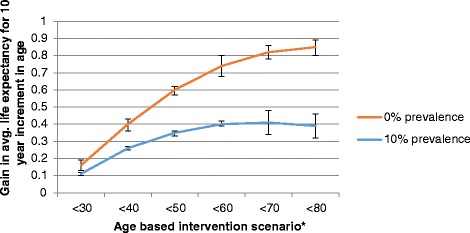
Overall gain in average life expectancy (ALE) for all men by intervention scenario. *For example, <40 indicates intervention to reduced smoking for all individuals aged <40 years

Figure 2 presents the corresponding gains in ALE for men, as summarised in Table 6. This shows a similar initial increase in ALE gain followed by a flattening of the effect. When smoking prevalence is reduced to 10 %, the plateauing effect again occurs at the point of intervening on persons aged <60. This again indicates that targeting smoking reduction in persons aged at least 60 years offers no additional gain in terms of population ALE. The gain in ALE for reducing smoking prevalence to 10 % for men aged <60 years is about 54 % of that obtained by complete cessation for that target intervention. When the entire population of men is targeted, the gain in ALE is 0.86 years if smoking is eliminated, compared to a gain in ALE of 0.39 years if prevalence is reduced to 10 %. Therefore, reducing prevalence to 10 % across the population achieves only 45 % of the maximum possible gain in ALE.

Table 7 summarizes the gains in ALE for female and male current smokers if they were to cease smoking or if prevalence were reduced to 10 %. Maximal gains if age was targeted to reduce smoking prevalence to 10 % are 2.04 years for men and 2.00 years for women, both for age group <80 years. However if smoking were to be eliminated completely the gains in ALE in smokers would be approximately 4.46 years for men and 5.27 years for women for this intervention. Thus, if smoking prevalence was reduced to 10 % by targeting the age groups, the maximal gains in ALE for men and women are about 46 % and 38 % respectively of the maximum gains achievable through complete smoking cessation.

**Table 7:** Gain in Average Life Expectancy (ALE) Amongst Smokers in the Population Under Various Smoking Reduction Scenarios (study period 2012-13)

Scenario	Gain in ALE relative to base scenario (95 % CI)	
	Men	Women
Base scenario	-	-
0 % prevalence <30 years	0.63(-0.66,1.72)	0.85 (0.79,0.91)
10 % prevalence <30 years	0.43 (0.39,0.47)	0.73 (0.61, 0.91)
0 % prevalence <40 years	1.63(1.46,1.75)	1.82 (1.61,1.93)
10 % prevalence <40 years	1.06 (1.02, 1.10)	1.12 (0.96, 1.18)
0 % prevalence <50 years	2.61 (2.48, 2.70)	2.84 (2.66, 2.96)
10 % prevalence <50 years	1.52 (1.44, 1.57)	1.54 (1.42, 1.66)
0 % prevalence <60 years	3.40 (2.85, 3.77)	3.74 (3.24, 4.32)
10 % prevalence <60 years	1.84 (1.79, 1.93)	1.84 (1.71, 1.97)
0 % prevalence <70 years	4.03 (3.83, 4.23)	4.52 (4.25, 4.94)
10 % prevalence <70 years	2.02 (0.49, 4.03)	1.99 (1.85, 2.12)
0 % prevalence <80 years	4.46 (4.19, 4.67)	5.27 (5.20, 5.35)
10 % prevalence <80 years	2.04 (0.63, 3.46)	2.00 (1.86, 2.15)
10 % prevalence in entire population	2.08 (1.92,2.30)	2.05 (-0.23, 4.48)
0 % prevalence in entire population	4.59 (3.84,5.34)	5.55 (5.47,5.62)

**Fig. 3:**
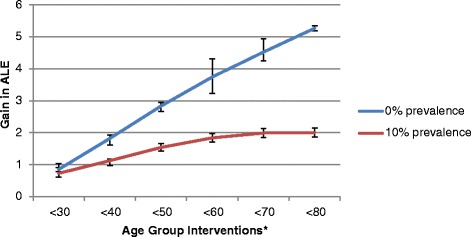
Overall gain in average life expectancy (ALE) for women smokers by intervention scenario. *For example, <40 indicates intervention to reduced smoking for women smokers aged <40 years

**Figs. 4:**
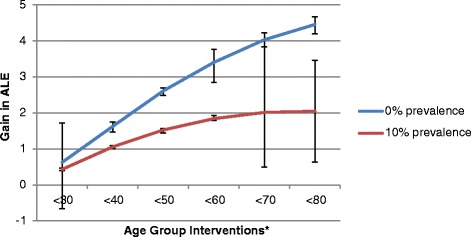
Overall gain in average life expectancy (ALE) for men smokers by intervention scenario. *For example, <40 indicates intervention to reduced smoking for men smokers aged <40 years

Figures 3 and 4 present the gain in ALE results for women and men smokers, as summarised in Table 7. For scenarios in which smoking prevalence is reduced to 10 %, the smallest gain in ALE occurs when only women less than 30 years are targeted (Fig. 3). As older age groups are included in the intervention scenarios the gain in ALE increases. However the gain in ALE begins to plateau as women greater than 70 years are included in the targeted population. A similar situation occurs for men who smoke (Fig. 4), with ALE continuing to increase as older age groups are included but slowing down when men aged at least 70 years are included in the targeted population.

## Discussion

In this study we evaluated the impact of several smoking prevalence reduction and cessation scenarios on average life expectancy in Australia, with interventions targeting increasingly older age groups. It was appropriate to relate the gains by a reduced smoking prevalence to 10 %, to the estimated gains in the case of total smoking cessation because the inclusion of complete cessation as a scenario allowed us to quantify the maximal gain in average life expectancy which can be used as a benchmark with which to compare gains from more realistic prevalence reduction targets.

We found that reducing the prevalence of smoking in the Australian population to 10 % could increase average life expectancy of all men by approximately 0.1 to 0.4 years, and all women by approximately 0.1 to 0.3 years. These are at best 54 % and 49 % for men and women respectively of the maximal life expectancy gains that could be achieved by complete smoking cessation. Amongst smokers the potential gains are greater, with an increase in average life expectancy amongst men smokers of approximately 0.4 to 2 years, and approximately 0.74 to 2 years amongst women smokers. However the gains in life expectancy for men and women smokers associated with 10 % smoking prevalence were at best 46 % and 38 % respectively of the maximal gains achievable through complete elimination of smoking. The maximal gains in life expectancy for men and women associated with 10 % smoking prevalence at the population level occurred when both aged less than 60 years were targeted. This indicates that including older people in the targeted population dilutes the effect of smoking reduction interventions, and therefore concentrating smoking cessation programs on populations aged less than 60 years represents a more optimal strategy.

As has been noted previously it is difficult to interpret gains in life expectancy from preventive interventions as any gains are usually averaged across an entire target population and may range from just weeks to months [13]. The target population will include individuals who gain little additional life expectancy as well as individuals who gain years of extra life. After reviewing 83 published reports of gains in life expectancy from a range of medical interventions, including both preventive measures and disease treatments, Wright and Weinstein [13] concluded that “a gain in life expectancy of a month from a preventive intervention targeted at populations at average risk and a gain of a year from a preventive intervention targeted at populations at elevated risk can both be considered large”. Since our estimated maximal gains in life expectancy to achieve a smoking prevalence of 10 % is at best 0.4 years for men and 0.3 years for women, for a population at an elevated risk of mortality such as Australia, the results we have obtained are consistent with potentially moderate or medium gains in life expectancy if smoking prevalence could be reduced to 10 %, in line with the Government set targets. The moderate gains in life expectancy at the population level are plausible given the already relatively “low” smoking prevalence in the population as the non-smokers dilute them. However, the gains are large when considering smokers only. In other settings or countries with higher baseline prevalence the gains would be larger at both the population level and for smokers.

Previous studies often defined smoking reduction as at least 50 % reduced from baseline at a population level without targeting of specific age or sex groups [14, 15]. In contrast, our study targets specific age groupings for each sex for reductions in smoking prevalence. Also, our targets are defined according to policies adopted by successive Australian governments to reduce smoking prevalence to 10 % by 2018. This is the first study in Australia which quantified improvements in survival due to reductions in smoking prevalence amongst different age and sex groups. It demonstrated a clear and positive gradient in life expectancy with increasing reduction in smoking prevalence in the Australian population.

We restricted our analysis to all-cause mortality as it is an objective endpoint and is available for all individuals in the AUSDIAB study. However, it is unclear whether the relative impact of the interventions we studied on mortality translates to other health outcomes such as major smoking related diseases and their associated disability burden. Further work is needed in this area.

Whilst the analysis was performed in a cross-sectional setting, the changes in the smoking behavior have primarily long-term effects. The life table method enabled us to estimate the long-term effect of smoking reduction on life expectancy, on the assumption that the mortality rate for each risk percentile which was estimated on the basis of all baseline risk factors (for the baseline scenario) and the simulated distribution of smoking having a prevalence of 10 % and the actual baseline distribution of other risk factors (for each intervention scenario), would continue for the rest of the life of each member of a risk percentile. We did not directly model the lag or delay in the health benefits of smoking cessation. However, if there is any lag or delay in health benefits of quitting, we do not anticipate that to be very significant at the population level.

Although it is possible to estimate the number of deaths prevented or reductions in mortality rate due to reductions in smoking prevalence using the simpler population attributable risk method, our research question was to estimate improvements in life expectancy for which the population attributable risk method alone is not sufficient as an additional method, for example, the life table method will also be required to estimate life expectancy using the mortality rate estimated by the population attributable risk method. Since the risk percentiles method already incorporates the life table, we can estimate life expectancy as a direct output.

We gradually targeted ages <30 years, <40 years and so on below higher ages to determine the age after which there is no further gain in life expectancy or there is a slowing down in the gain. Performing the analyses for ages 30+, 40+ and so on would not have allowed us to achieve this objective (and so were not performed in this study). Also, targeting the population by below a certain age would be helpful to target fewer people compared to targeting the entire AUSDIAB sample based on the full age range of 25-80 + .

We targeted the current smokers to quit smoking in order to reduce smoking prevalence to 10 %. The issue of ex-smokers having higher risk of mortality than never smokers could not be modelled because the EURO SCORE [8] did not make any distinction between ex-smokers and never smokers while estimating risk scores as the risk equation which was used to estimate these had only two categories for smoking-current smokers and nonsmokers (nonsmokers included both ex-smokers and never smokers). In effect, the mortality rates among never and former smokers were averaged. The inability to distinguish between never and former smokers while estimating absolute risk of individuals is a limitation of this study. Despite this limitation of the EURO SCORE, it was found to recalibrate well to the AUSDIAB sample [9].

Since the smoking prevalence estimated using the 1999-2000 (baseline) AUSDIAB survey is approximately 16 %, the projected target of 10 % by 2018 seems superficially achievable. However, using dynamic forecasting modelling Gartner et al. [16] found that as the initiation rate has been declining in Australia, to achieve 10 % prevalence by 2020, the current cessation rate should be doubled. However, latest estimates in Australia indicate that the smoking rate among adults aged 14 years and over was 12.8 % and for 18 years and over was 13.3 % in 2013 [17]. There was a statistically significant decline between 2010 and 2013-the period during which the government target to achieve 10 % prevalence by 2018 was well in force. With 5 years remaining to reach 2018 since 2013, the target of achieving 10 % prevalence by 2018 seems to be achievable.

Since we have shown that 10 % smoking prevalence gives at best half of maximum possible gains under the scenario of complete cessation, further modelling will help to answer questions such as whether reduction of smoking prevalence to 5 % will remediate most of, or little of, the differential between reduction to 10 % prevalence and complete cessation. To achieve a target of 5 % we modelled for men and women gains in life expectancy for a few selected scenarios (results not shown), like the random reduction to a smoking prevalence of 5 % for the whole population and random reduction to 5 % prevalence for those below 60 years, and found that there was almost a proportionate decrease in gains in life expectancy compared to the corresponding scenario with a target of 10 % prevalence. The potential gains for men and women were at best 0.63 and 0.5 years which are 73 % and 68 % respectively of that obtained by complete smoking cessation. Thus, setting a target of 5 % prevalence would remediate most of the differential between reduction to 10 % prevalence and complete cessation. The analysis and issues presented in this paper are highly complex and as is the case in all scientific endeavours, there is always a possibility that methodological problems may affect the results and interpretation of the findings. One major concern is obviously the cross-sectional approach.

## Conclusion

This study suggests that smoking cessation programs aimed at reducing smoking prevalence in the Australian population to 10 % could have a moderate or medium impact on increasing average life expectancy, but a large impact on smokers average life expectancy The target of 10 % smoking prevalence offers at best 54 % of the potential life expectancy gains that could be achieved by complete elimination of smoking.

### Ethics

The study did not include any participant and only secondary data analysis was performed. Therefore no ethics approval was required.

## Additional file

Additional file 1:
**Using risk percentiles method for scenario modelling [** 18 **–** 22 **].** (DOC 1240 kb)
